# SLURP2 Enlarges Adipocytes and Induces IL‐23‐Producing Macrophages in Murine Dermal Adipose Tissue

**DOI:** 10.1111/exd.70304

**Published:** 2026-07-06

**Authors:** Hitomi Tsuji, Tomotaka Mabuchi

**Affiliations:** ^1^ Department of Dermatology Tokai University School of Medicine Isehara Kanagawa Japan

**Keywords:** adipocyte, cholesterol, enlargement, IL‐23‐producing macrophage, SLURP2

## Abstract

Dermal adipocytes have unique features different from visceral and subcutaneous adipocytes. Their cyclic remodelling is coupled with hair follicle (HF) cycling: expansion in the anagen and reduction in the catagen stages. Secreted Ly6/uPAR related protein 2 (SLURP2) is a non‐neuronal transmitter and a ligand of both nicotinic and muscarinic acetylcholine receptors. It is upregulated in psoriatic lesions. Mouse *SLURP2*‐deficient mice exhibit reduced adiposity and plasma cholesterol levels, in addition to palmoplantar keratoderma. However, the effects of SLURP2 on adipocytes remain unexplored. Here, we demonstrated that SLURP2 regulates murine dermal adipocyte size independently of HF cycling and upregulates interleukin (IL)‐23 gene expression following intradermal injection of recombinant human SLURP2‐Fc protein (rSLURP2) and phosphate‐buffered saline (control) at different dorsal sites of C57BL/6J mice after depilation. SLURP2 enlarged dermal adipocytes independently of HF cycles by inhibiting sterol regulatory element‐binding protein‐1, a transcription factor involved in cholesterol and fatty acid synthesis, which leads to the activation of cholesterol synthesis followed by expansion of the plasma and lipid droplet membrane. Immunohistochemical staining of paired mirror sections revealed a statistically significant increase in the ratio of IL‐23‐producing macrophages in the dermal adipose tissue of rSLURP2‐injected skin compared with the control. Furthermore, IL‐23p19 gene expression was significantly upregulated in rSLURP2‐treated skin. Taken together with the previous findings, the present study showed that SLURP2 has inflammatory and metabolic effects. Dermal adipose tissues may serve as a source of IL‐23 in psoriatic lesions.

## 1 Introduction

Dermal white adipose tissue (dWAT) is located beneath the reticular dermis in the skin [1]. In mice, the *panniculus carnosus*, which is rudimentary in humans, clearly divides dWAT from subcutaneous white adipose tissue (sWAT), whereas in humans, dWAT is connected with sWAT. In addition, dWAT is adjacent to hair follicles (HFs). dWAT has unique features which are distinct from sWAT and visceral white adipose tissue [2]. The most characteristic feature is to remodel periodically in coordination with the HF cycle [2]. dWAT cycles the expansion in the anagen stage and the reduction in the catagen stage [2]. Dermal adipocytes repeat the cellular conversions: mature dermal adipocytes de‐differentiate into the much smaller fibroblast‐like adipocyte‐derived preadipocytes (ADP) in the catagen stage and re‐differentiate back into mature adipocytes in the anagen stage [3]. De‐differentiated dermal adipocytes release lipids into the reticular dermis, which can connect in the skin inflammation [3]. Re‐differentiated dermal adipocytes secret cathelicidin, an antimicrobial peptide, which attracts neutrophils, monocytes, T cells and mast cells and exerts a proinflammatory effect [4]. Cathelicidin is upregulated in human psoriatic skin [5].

SLURP2, a secreted Ly6/uPAR related protein 2, is a non‐neuronal ligand of acetylcholine receptors (AChRs) [6]. It binds to various types of AChRs, including α2, α4–α7, α9, β2, β4 and M1–5 [6, 7, 8]. SLURP2 was first identified in the skin [9] and is expressed in many organs, including the esophagus, stomach, duodenum, colon, as well as in epidermal and oral keratinocytes, mononuclear leukocytes, dendritic cells and macrophages [6, 9, 10]. Previous studies have revealed several effects of SLURP2 in oral or epidermal keratinocytes and immune cells: (1) delayed differentiation, (2) inhibition of terminal keratinization, (3) prevention of apoptosis in keratinocytes and (4) anti‐inflammatory effects in T cells and macrophages [6, 11]. However, its effects on adipocytes remain unexplored. Importantly, Lyukmanova et al. elucidated the following pharmacological features of SLURP2 using electrophysiological methods and computer modeling: (1) SLURP2 inhibits ACh‐evoked currents at α3β2, α4β2 and α7 nicotinic AChRs (nAChRs) at high concentrations and (2) it can bind to the classical orthosteric agonist/antagonist binding sites at α7 and α3β2 nAChRs [8]. These findings indicate that SLURP2 can exert both anti‐inflammatory and pro‐inflammatory effects depending on its concentration and binding site. However, no reports confirm the latter effect in vivo.

By contrast, mouse *SLURP2*‐deficient mice exhibit not only palmoplantar keratoderma but also metabolic phenotypes, including reduced adiposity, loss of body weight and decreased plasma cholesterol levels [12]. These results indicate that SLURP2 is involved in metabolism. However, the previous study did not explore the underlying mechanisms of these metabolic phenotypes [12].

Here, we elucidated this unexplored mechanism through intradermal injection of recombinant SLURP2 (rSLURP2) and PBS (control) at different sites on the murine back. Furthermore, we revealed that SLURP2 stimulates IL‐23 expression in murine skin and induces IL‐23‐producing macrophages in dermal adipose tissues. SLURP2 is upregulated in psoriatic lesions [9]. Taken together with previous findings, the present study suggests that SLURP2 may be involved in both inflammation and metabolism in the pathophysiology of psoriasis.

## 2 Materials and Methods

### 2.1 Mice

Six‐ or 10‐week‐old male C57BL/6 mice were purchased from Charles River (Tokyo, Japan) and maintained under a 12‐h light/12‐h dark cycle with free access to a normal chow diet and water in a conventional facility until 18 weeks of age for all experiments. All experimental protocols were approved by the Animal Experimentation Committee of Tokai University (approval numbers: 211095 in 2021, 222027 in 2022, 233009 in 2023 and 244013 in 2024). All animal procedures were performed in accordance with the animal experiment guidelines of the Japan Science Council.

### 2.2 Experimental Design

**FIGURE 1:**
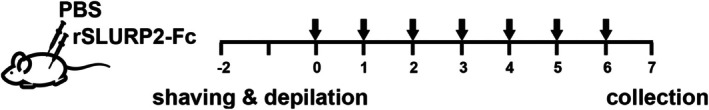
*Experimental design*. The mouse's back hair was shaved followed and depilated 2 days before the experiment. Five hundred μg / 50 μL of recombinant human SLURP2‐Fc protein and 50 μL PBS were intradermally injected into different sites on the back of each male C57BL/6J mouse every 24 h for 7 days. On the 8th day (7th day in the figure), each injected skin sample was collected. *Abbreviations*: rSLURP2‐Fc, recombinant secreted Ly‐6/uPAR related protein 2 fused with Fc; PBS, phosphate‐buffered saline.

The entire backs of the mice were shaved with an electric shaver followed by depilation with a depilatory cream 2 days before the experiment. Recombinant human SLURP2 Fc Chimera protein (rSLURP2) (10035‐SP, R&D Systems, Minneapolis, MN, USA) was prepared at a concentration of 500 ng/50 μL in phosphate‐buffered saline (PBS) and 50 μL of this solution or PBS alone was injected intradermally into different dorsal sites once daily for 7 days (Figure 1). To confirm that the Fc protein did not affect the results, three mice were intradermally injected with 500 ng/50 μL of recombinant human IgG1 Fc (rFc) (110‐HG, R&D Systems) at dorsal sites separate from those injected with rSLURP2 and PBS. On the 8th day, mice were euthanized using 50% isoflurane. Each injected skin sample was collected for analysis.

### 2.3 Histological Analyses

Skin samples injected with rSLURP2‐, rFc‐ or PBS were fixed in 10% formalin and embedded in paraffin. After deparaffinization and hydration, 5 μm‐thick sections were stained with haematoxylin and eosin (H&E).

### 2.4 Measurement of Adipocyte Areas

Images of H&E‐stained sections were captured at 100× magnification using BX63 microscopes (digital camera DP74, 75; Olympus Optical, Tokyo, Japan). The areas of individual adipocytes in dermal adipose tissues of each section were measured using Fiji software [13]. The mean area was calculated as follows: each sum of the adipocyte areas was divided by the total numbers of counted adipocytes (Table S1). The effects of SLURP2 and PBS were compared in each mouse, and adipocytes were categorized by size to calculate their proportional distribution. In addition, the mean adipocyte size was compared among the SLURP2, Fc and PBS groups in three mice.

### 2.5 Identification of HF Stages in Dermal Adipose Tissues

Using images of the aforementioned H&E‐stained sections, the hair cycle stage of each HF in dermal adipose tissues or in the dermis was identified according to the classification of murine HFs by Müller‐Röver S et al. [14]. To compare the hair cycle phase between the SLURP2 and control groups, the hair cycle score was calculated as described previously [14]. In brief, every stage of anagen was assigned the number of the stage (e.g., anagen I = 1, anagen II = 2, anagen III = 3, etc.). Each mean HF stage was calculated by dividing each sum by the total number of counted HFs.

### 2.6 Immunohistochemical Analyses

To identify IL‐23‐producing macrophages in the dermal adipose tissue, paired mirror sections (3‐μm thickness) were prepared. After deparaffinization and hydration, antigen retrieval pretreatment was performed using Target Retrieval Solution (pH 6.0; S1699; Agilent DAKO, Santa Clara, CA, USA) at 121°C for 10 min. Endogenous peroxidase activity was quenched in 0.3% hydrogen peroxide for 30 min, followed by blocking with PBS containing 10% normal goat serum and 1% bovine serum albumin for 1 h at 25°C. Each section of a mirror pair was incubated overnight at 4°C with either rat anti‐mouse F4/80 antibody (clone A3‐1; MCA497, 1:200; Bio‐Rad Laboratories, Hercules, CA, USA) or IL‐23p19 polyclonal antibody (PA5‐20239, 1:500; Invitrogen, Carlsbad, CA, USA). The sections were treated with rat horseradish peroxidase (HRP) polymer and rabbit alkaline phosphatase (AP) polymer using the DoubleStain IHC Kit (Green/HRP & AP/Red, ab183285; Abcam, Cambridge, UK) for 30 min, according to the manufacturer's instructions. The sections were then stained with Emerald Green or Fast Red using the same kit. Counterstaining was not performed. Moreover, immunohistochemical staining was performed on 5‐μm sections using 5 μg/mL sterol regulatory element‐binding protein (SREBP) 2 antibody (#NBP2‐41282; Novus Biologicals, Centennial, CO, USA), Caveolin (Cav)‐1 (D46G3) rabbit monoclonal antibody (#3267, 1:400; Cell Signalling Technology, Danvers, MA, USA) and N‐Histofine Simple Stain mouse MAX‐PO (R) (#414141F; Nichirei Biosciences Inc., Tokkyo, Japan) as aforementioned, except 98°C antigen retrieval was used for Cav‐1. Sections were stained with 3,3′‐diaminobenzidine and counterstained.

### 2.7 Counting IL‐23‐Producing Cells, Macrophages and IL‐23‐Producing Macrophages in the Dermal Adipose Tissue

Images of immunostained mirror‐pair sections at 200× magnification were captured using a BX63 microscope. When image brightness was low, the brightness and contrast of the entire image were uniformly adjusted using Fiji software to ensure accurate counting. IL‐23p19‐ or F4/80‐positive cells were first counted in each mirror‐section pair using Fiji software. Double‐positive cells identified in the mirror‐section pair were counted as IL‐23‐producing macrophages. Then, the ratio of IL‐23‐producing macrophages to total macrophages (F4/80‐positive cells) in the dermal adipose tissue was calculated.

### 2.8 Quantitative Analysis of Immunohistochemical Staining of Cav‐1‐Positive Dermal Adipocytes

Images of immunostained 5‐μm sections at 200× magnification were captured using a BX63 microscope. Staining intensity was divided into four levels and scored as follows: no stain, 0; weak staining, 1; moderate staining or strong staining on less than half of the membrane, 2; and strong staining on the entire membrane, 3. After colour‐split of each image using Fiji software, blue split sections were used. Each adipocyte in dermal adipose tissues was scored, and each mean staining score was calculated by dividing each total score by the counted adipocytes number.

### 2.9 Counting SREBP‐2‐Positive Adipocytes in the Dermal Adipose Tissue

Images of immunostained 5‐μm sections at 400× magnification were captured using a BX63 microscope. Dermal adipocytes showing any level of staining were counted as SREBP‐2‐positive. Total dermal adipocytes and SREBP‐2‐positive adipocytes were counted using Fiji software. The ratio of SREBP‐2‐positive adipocytes to total dermal adipocytes was calculated.

### 2.10 RNA Isolation and Real‐Time Quantitative Reverse Transcription Polymerase Chain Reaction (qRT‐PCR) Analyses

Total RNA was isolated from rSLURP2‐, rFc‐ or PBS‐injected mouse skin using the RNeasy Fibrous Tissue Mini Kit (74704; Qiagen, Hilden, Germany), according to the manufacturer's instructions. A total of 5 or 25 ng of RNA was reverse transcribed into complementary DNA and amplified using TaqMan Gene Expression Assays (ThermoFisher, Waltham, MA, USA) and the THUNDERBIRD Probe One‐Step qRT‐PCR Kit (QRZ‐101; Toyobo Co. Ltd., Osaka, Japan) with Uracil‐DNA Glycosylase (UNG), heat‐labile (UNG‐101; Toyobo Co. Ltd.), following the manufacturer's protocol. Real‐time RT‐PCR was performed on an ABI QuantStudio 3 Real‐Time PCR System (Applied Biosystems, Carlsbad, CA, USA). Quantification was performed using the standard curve method. Mouse C57 skin total RNA (MR‐101‐C57; Zyagen, San Diego, CA, USA) was used to construct standard curves. All samples were analysed in parallel for 18S ribosomal RNA (rRNA) expression as an internal control, and messenger RNA (mRNA) expression was normalized to that of 18S rRNA. The coefficients of determination (R^2^) for standard curves were as follows: c‐c motif chemokine ligand 2 (ccl2), nitric oxide synthase (NOS) 2 (iNOS), Arginase (Arg) −1 and 18S rRNA—R^2^ = 0.999; il23a and 18S rRNA—R^2^ = 0.999 and 0.996, respectively; and Srebf2 and 18S rRNA—R^2^ = 0.998 and 1, respectively. TaqMan Gene Expression Assays were performed using the following probes: Mm00518984_m1 IL23a (FAM), Mm00441242_m1 Ccl2 (FAM), Mm00440502_m1 Nos2 (FAM), Mm00475988_m1 Arg1 (FAM), Mm01306292_m1 Srebf2 (FAM), Mm03928990_g1, Rn18S and Rn45S (VIC).

### 2.11 Simple Western Blotting Analysis

Total protein was extracted from rSLURP2‐, rFc‐ or PBS‐injected mouse skin using the Minute Total Protein Extraction Kit for Skin Tissue (SA‐01‐SK; Invent Biotechnologies Inc., Plymouth, MN, USA) according to the manufacturer's protocol. Protein concentrations were determined using the DC Protein Assay (5000112; Bio‐Rad Laboratories). Western blotting was performed using the Protein Simple Wes system (ProteinSimple, San Jose, CA, USA) with a 12‐230 kDa separation module (SM‐W004) and anti‐rabbit detection module (DM‐001A), following the manufacturer's instructions. The following primary antibodies were used: peroxisome proliferator‐activated receptor (PPAR) γ (81B8) Rabbit mAb (#2443; Cell Signalling Technology) (1:50); CCAAT/enhancer‐binding protein (C/EBP) α (D56F10) XP Rabbit mAb (#8178; Cell Signalling Technology) (1:50); platelet‐derived growth factor (PDGFR) β (28E1) Rabbit mAb (#3169; Cell Signalling Technology) (1:10); and SREBP1 Antibody (#NB100‐2215; Novus Biologicals) (1:25). The Anti‐Rabbit Secondary HRP Antibody from the detection module was used as the secondary antibody. Simple Western blot data were visualized and quantified using Compass for SW software (version 6.1.0; ProteinSimple, San Jose, CA, USA). The peak that matched the molecular weight of the target gene was selected, that is, the highest peak. A shift in apparent molecular weights of ±10% was accepted. Target protein expression was normalized to the total amount of protein detected using the Total Protein Detection Module (DM‐TP01A; ProteinSimple).

### 2.12 Statistical Analyses

Quantitative differences among the three groups were assessed using the Friedman test, followed by the Dunn's multiple comparison test. Differences between the two groups were analysed using the Wilcoxon matched‐pairs signed‐ranks test. Statistical analyses were performed using GraphPad Prism (version 10.4.1; GraphPad Software, San Diego, CA, USA), and values of *p* < 0.05 (*) were considered statistically significant.

## 3 Results

### 3.1 Results of Recombinant Human SLURP2 Fc Chimera Protein Are due to SLURP2, Not Fc

To confirm the absence of Fc‐specific effects in rSLURP2, we examined statistical differences among the three groups in the mean adipocyte area and IL‐23‐producing macrophage ratio using the Friedman test followed by Dunn's correction. Given that no significant Fc‐specific effects were observed (Figure S1), we confirmed that the observed effects of rSLURP2 were due to SLURP2, and not Fc. Consequently, PBS was used as the control. No other Fc experiments were performed, except for histological and immunohistochemical analyses.

### 3.2 SLURP2 Enlarges Dermal Adipocytes

**FIGURE 2:**
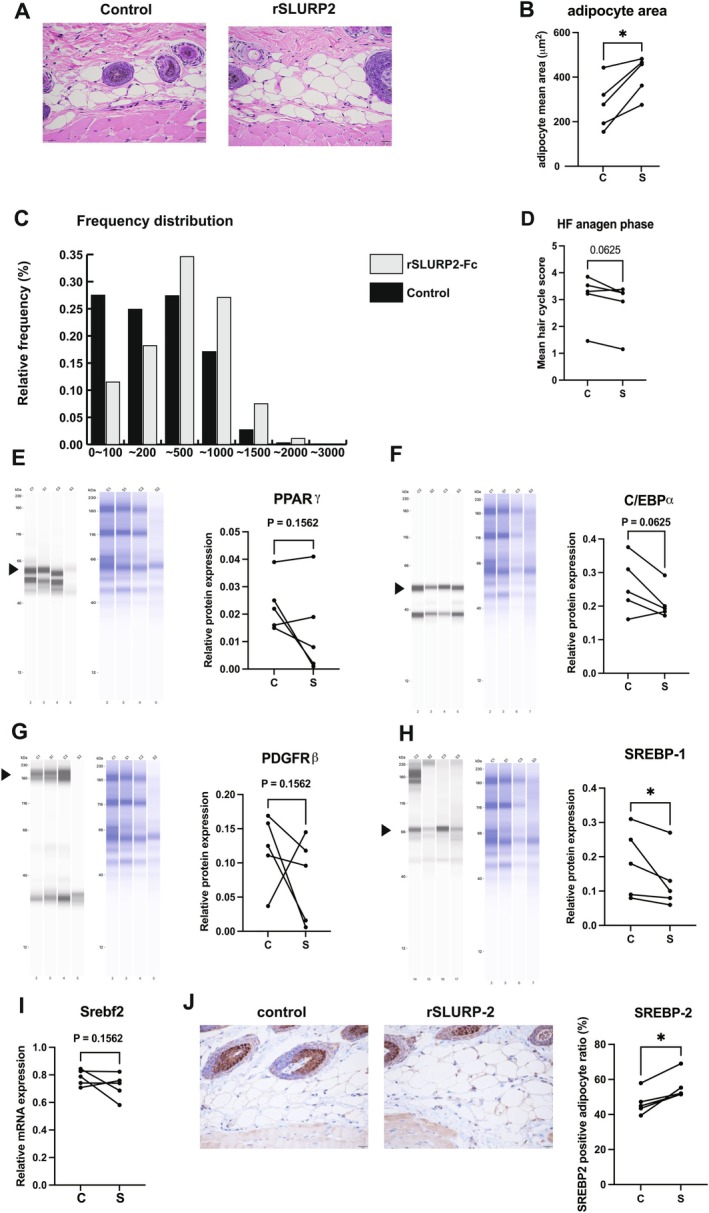
*SLURP2 induces the enlargement of dermal adipocytes by inhibiting SREBP‐1*. (A) Representative images (×200) of H&E stained sections of the dermal adipose tissue in rSLURP2 or control (PBS)‐injected back skin for 7 days. Scale bar: 50 μm. (B) Comparison of mean area and (C) size distribution of dermal adipocytes in rSLURP2‐ or control‐injected back skin (*n* = 5). (D) Comparison of anagen phase in rSLURP2‐ or control‐injected back skin for 7 days (*n* = 5). (E‐G) Relative protein expression of the transcription factors PPARγ, C/EBPα, PDGFRβ and (H) SREBP‐1 in rSLURP2‐ or control‐injected back skin (*n* = 5). Representative lanes/images for the target and total protein. They were selected from five mice. Some representative lanes/images for total protein have been used repeatedly, with the corresponding lane/sample labels clearly indicated. Black arrows indicate the molecular weight of each target gene. (I) Relative gene expression of the transcription factor Srebf2 in rSLURP2‐ or control‐injected back skin (*n* = 5). (J) Representative images of immunohistochemical staining for SREBP‐2 (×400) in dermal adipose tissues of rSLURP2‐ or control‐injected back skin, and comparison of SREBP‐2‐positive ratio in dermal adipocytes between rSLURP2‐ and control (*n* = 5). Scale bar: 20 μm. Data were analysed by Wilcoxon matched‐pairs signed‐ranks test for panels B, D–H. *p < 0.05 versus control. *Abbreviations*: H&E, haematoxylin and eosin; PPARγ, peroxisome proliferator‐activated receptor gamma; C/EBPα, CCAAT/enhancer‐binding protein alpha; PDGFRβ, platelet‐derived growth factor receptor beta; SREBP, sterol regulatory element‐binding protein; Srebf2, sterol regulatory element‐binding transcription factor 2; rSLURP2, recombinant secreted Ly‐6/uPAR related protein 2 fused with Fc; PBS, phosphate‐buffered saline.

No obvious skin changes were observed at either the rSLURP2‐ or control‐injected sites. We also examined histological changes, and the histological analysis showed enlargement of dermal adipocytes in rSLURP2‐ injected skin compared with the control (Figure 2A). The mean adipocyte area in the SLURP2 group was significantly larger than that in the control group (Figure 2B). Furthermore, the data were grouped by adipocyte size, and the proportion of each size category was calculated. The frequency distribution showed that the ratios of adipocytes > 200 μm^2^ (the lowest area in the highest frequency of the two) were 70% in the SLURP2 group and 48% in the control group (Figure 2C).

### 3.3 HF Growth Phase Shows no Significant Difference Between SLURP2‐ and Control‐Injected Dermal Adipose Tissues

Dermal adipocytes coordinate with the hair cycle to remodel, that is, anagen‐coupled expansion called re‐differentiation, and catagen‐coupled regression called de‐differentiation [2]. We induced the telogen‐anagen follicle stage by depilation. Skin colour has been demonstrated to be consistent with the HF stages: pink in the telogen stage, from grey to black in the anagen stage and black in the catagen stage [14]. Given that the back skin colour of three mice was uniformly pink and that of the other two was white on the Day 0, we judged the HF stage at the starting point of the experiment before injection as the telogen stage. To examine whether SLURP2 is involved in hair cycle growth, hair cycle stages of all HFs in rSLURP2‐ and control‐injected dermal adipose tissues were identified using images of H&E‐stained sections. Anagen III was the most common of all anagen phases in both rSLURP2‐ and control‐injected dermal adipose tissues. According to the timescale for the hair cycle after depilation by Müller‐Röver S et al., anagen stages last at least 2 weeks and catagen stages begin at approximately Day 18 [14]. Our results are almost consistent with their timescale.

Furthermore, one mouse exhibited no HFs in both dermal adipose tissues since anagens I and II were occupied in both the dermis except for anagen III at the border of the dermal adipose tissue in the control (Figure S2). The mean HF cycle score showed no significant difference between the SLURP2 and control groups (Figure 2D).

### 3.4 SLURP2 Increases Protein Expression of Cav‐1 in Dermal Adipocytes Compared With the Control

Caveolin‐1 is a constituent of caveolae, invagination of the plasma membrane, that binds cholesterol [15]. Given that adipocyte expansion correlates with Cav‐1 expression [16], we examined Cav‐1 protein expression using immunohistochemical staining (Figure S3A). The mean staining score was significantly higher in the SLURP2 group than in the control group (Figure S3B).

### 3.5 PPARγ, C/EBPα and PDGFRβ Are Not Involved in the Enlargement of Adipocytes Caused by SLURP2

To clarify how SLURP2 regulates dermal adipocyte size, we examined the expression of transcription factors related to adipogenesis: PPPARγ, C/EBPα and PDGFRβ, using simple Western blotting analysis. The first two are key transcription factors that regulate terminal differentiation during white adipogenic progression [17, 18], while the third is a negative regulator of adipogenesis that inhibits adipocyte differentiation [19]. Mature adipocytes express PPARγ and C/EBPα as well as preadipocytes [18]. These transcriptional factors are involved in lipogenesis. PPARγ agonist activates adipose tissue lipolysis [20] and increases the number of small adipocytes in obese rats [21]. By contrast, downregulation of these transcription factors has been shown to cause lipid accumulation in differentiated adipocytes, leading to their enlargement [22]. Our results showed no statistically significant differences in the expression of these adipogenic transcription factors between the rSLURP2 and control groups (Figure 2E–G).

### 3.6 SLURP2 Downregulates SREBP‐1

Next, we examined other transcription factors involved in lipogenesis, namely the SREBPs. Adipocytes not only take up but also synthesize cholesterol and triglycerides (TG) [23]. SREBPs consist of SREBP‐1 and ‐2. The former has two isoforms, SREBP‐1a and ‐1c. SREBP‐1 mainly regulates free fatty acid (FFA) synthesis, which provides substrates for TG formation, while SREBP‐2 is the master regulator of cholesterol synthesis [24]. Their interactions during cholesterol synthesis have been well demonstrated [25]. Given that mouse *SLURP2*‐deficient mice exhibit decreased plasma total cholesterol [12], we hypothesized that SLURP2 would be involved in cholesterol synthesis in dermal adipocytes. SLURP2 treatment significantly reduced the protein expression of SREBP‐1 compared with the control (Figure 2H).

### 3.7 Ratio of SREBP‐2‐Positive Adipocytes in Dermal Tissue Is Significantly Higher in the SLURP2 Group Than in the Control

The gene expression of *Srebf*2 showed no significant difference between the rSLURP2 and control groups (Figure 2I). Since we could not assess protein expression due to the lack of a suitable antibody for simple Western blotting analysis or a sufficient protein for conventional Western blotting analysis, we compared the ratio of SREBP‐2‐positive adipocytes in dermal adipose tissue between the rSLURP2 and control groups. This ratio was significantly higher in the rSLURP2 group than in the control group (Figure 2J).

### 3.8 SLURP2 Upregulates the mRNA Expression of IL‐23p19

**FIGURE 3:**
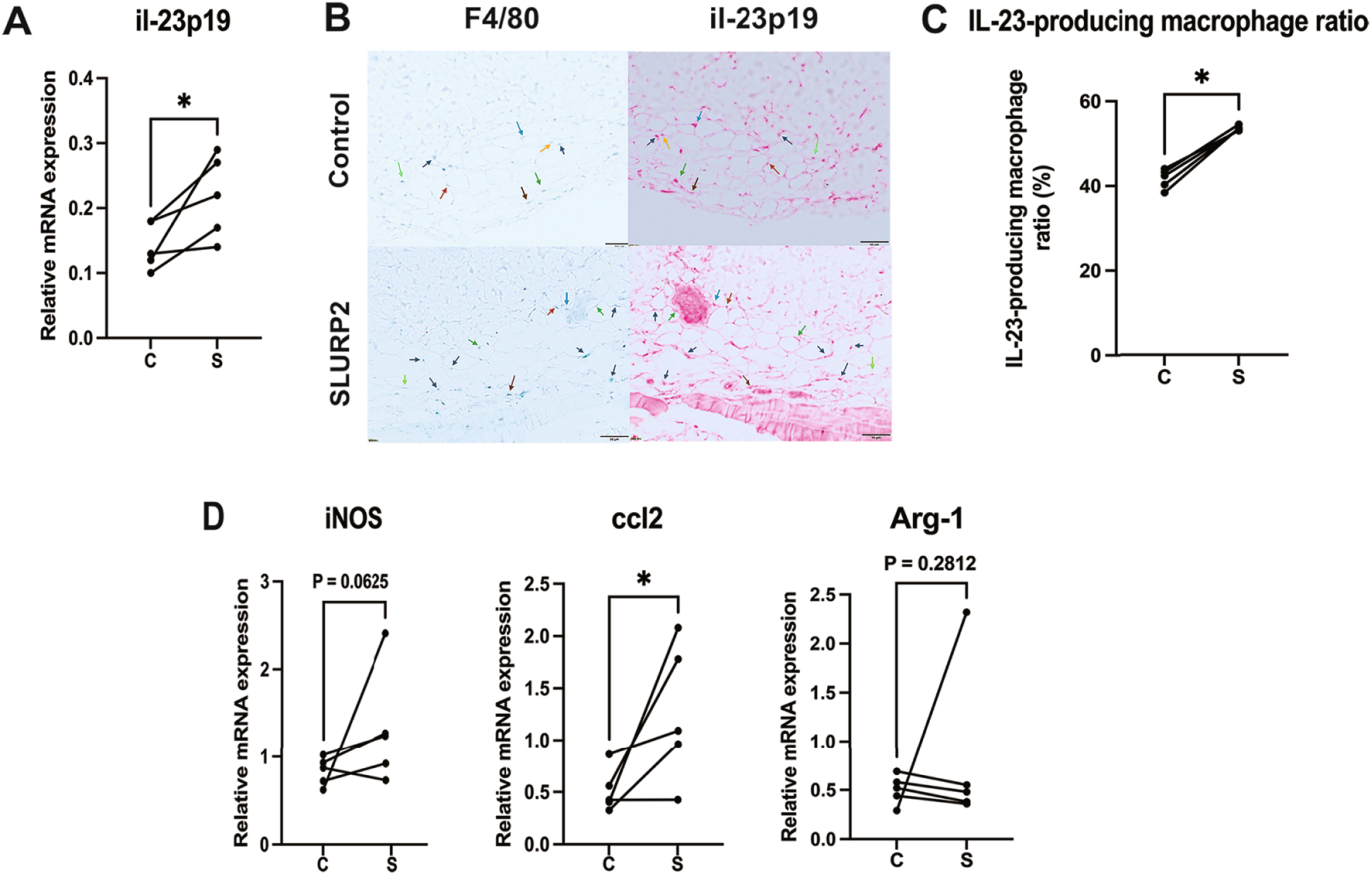
*SLURP2 induces IL‐23‐producing macrophages*. (A) Gene expression of IL‐23p19 in rSLURP2 or control‐injected back skin (*n* = 5). (B) Representative images of immunohistochemical staining for F4/80 and IL‐23p19 (×200) in dermal adipose tissues using mirror‐pair sections. Arrows of the same colour indicate IL‐23‐producing macrophages. Scale bar: 50 μm. (C) Comparison of IL‐23‐producing macrophage proportion in dermal adipose tissues of rSLURP2 or control‐injected back skin (*n* = 5). (D) Gene expression of iNOS, ccl2 and Arg‐1 in rSLURP2 or control‐injected back skin for 7 days (*n* = 5). Data were analysed by Wilcoxon matched‐pairs signed‐ranks test for A, C and D. *p < 0.05, versus control. *Abbreviations*: IL, interleukin; iNOS, inducible nitric oxide synthase; ccl2, c‐c motif chemokine ligand 2; Arg‐1, arginase‐1; rSLURP2, recombinant secreted Ly‐6/uPAR related protein 2; PBS, phosphate‐buffered saline.

To elucidate how SLURP2 contributes to the pathophysiology of psoriasis, we examined the mRNA expression of IL‐23p19 using qRT‐PCR. IL‐23 is a key cytokine in the IL‐23/Th17 axis involved in the pathogenesis of psoriasis [26]. Our results showed that SLURP2 significantly upregulated the mRNA expression of IL‐23p19 compared with control (Figure 3A).

### 3.9 SLURP2 Induces IL‐23‐Producing Macrophages in the Dermal Adipose Tissue

Adipose tissue is in an anti‐inflammatory state in lean individuals, and this is characterized by the predominance of M2 macrophages and M2 polarization [27]. Given that SLURP2 was demonstrated to upregulate IL‐23 gene expression, we examined the proportion of IL‐23‐producing macrophages in the dermal adipose tissue, as M1 macrophages secret IL‐23. We identified IL‐23‐producing cells, macrophages and IL‐23‐producing macrophages in dermal adipose tissue using immunohistochemical analysis with mirror‐section pairs. We used F4/80 as a macrophage marker, and cells double‐positive for F4/80 and IL‐23p19 were identified as IL‐23‐producing macrophages (Figure 3B). Subsequently, the ratio of IL‐23‐producing macrophages to total F4/80‐positive macrophages was calculated. The ratios were > 50% in the rSLURP2 group and 40% in the control group, and this difference was statistically significant (Figure 3C).

### 3.10 SLURP2 Upregulates the Gene Expression of ccl2, but Not iNOS

Next, to verify whether IL‐23‐producing macrophages were M1 macrophages, we assessed the mRNA expression of M1 macrophage markers (ccl2 [MCP‐1: monocyte chemoattractant protein‐1] and iNOS) and the M2 macrophage marker (Arg‐1) using qRT‐PCR. Given that iNOS gene expression showed no statistically significant differences between SLURP2 and the control, we could not identify IL‐23‐producing macrophages as M1 macrophages. Additionally, SLURP2 did not significantly induce the downregulation of the M2 macrophage marker, Arg‐1. However, it significantly upregulated ccl2 mRNA expression compared with the control (Figure 3D).

## 4 Discussion

In the present study, we demonstrated for the first time that SLURP2 enlarges murine dermal adipocytes independently of HF cycles. In addition, we found that SLURP2 upregulates the expression of IL‐23 in the skin and induces IL‐23‐producing macrophages in murine dermal adipose tissues. Overall, our results indicate that SLURP2 exerts both proinflammatory and metabolic effects.

The metabolic function of SLURP2 has been extensively described in a previous study by Allan et al., who showed that mouse *SLURP2*‐deficient mice exhibited metabolic phenotypes and reduced adiposity. However, the underlying mechanism has not yet been explored. This article further suggested that increased grooming of pads may have been the cause of this phenomenon [12], and in the present study, we elucidated this mechanism. Our results indicate that the reduction in adiposity may result from decreased adipocyte size caused by mouse *SLURP2* deficiency. Accordingly, together with the results of the previous study, our findings suggest that SLURP2 is involved in the regulation of adipocyte size and contributes to lipid homeostasis.

Given that dermal adipocyte size is affected by the HF cycle, with expansion in the anagen and reduction in the catagen stages [2], we examined whether SLURP2 affects the HF cycle. Our results revealed no statistically significant difference in HF stages between SLURP2 and control. Notably, one mouse exhibited no HF in the dermal adipose tissues due to anagen I and II phases (Figure S2). Accordingly, these results indicate that SLURP2 enlarges dermal adipocytes independently of the HF cycle.

**FIGURE 4:**
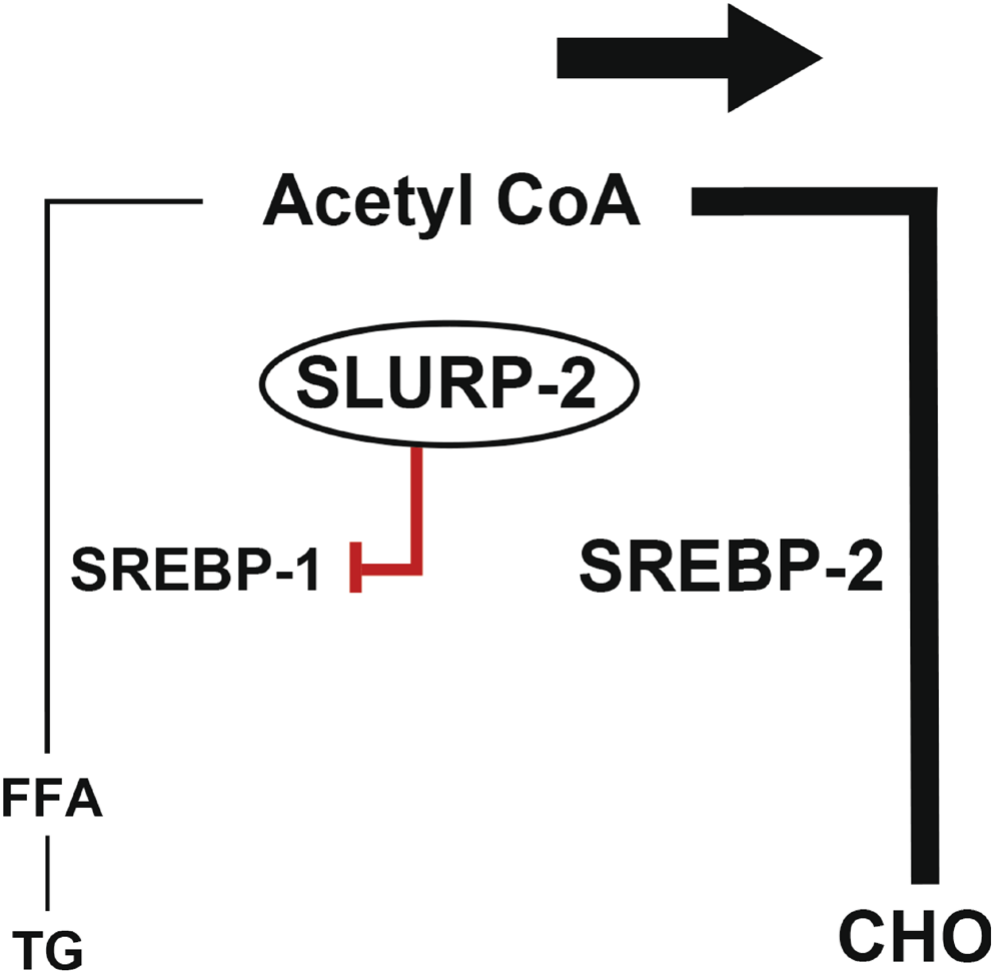
*The mechanism of cholesterol synthesis regulated by SLURP2*. Schematic illustration of the mechanism of cholesterol synthesis regulated by SLURP‐2. *Abbreviations*: SLURP2, secreted Ly‐6/uPAR related protein 2; SREBP, sterol regulatory element‐binding protein; FFA, free fatty acid; TG, triglyceride; CHO, cholesterol.

To further explore how SLURP2 enlarges adipocytes, we subsequently examined three adipogenic transcription factors: PPARγ, C/EBPα and PDGFRβ, finding no statistically significant differences between the SLURP2 and control groups (Figure 2E–G). Notably, mouse *SLURP2*‐deficient mice exhibited a significant decrease in serum cholesterol levels [12]. Adipocyte cholesterol correlates positively with adipocyte size [28]. Accordingly, we hypothesized that SLURP2 would be involved in cholesterol synthesis. This pathway has been reported to be regulated by the interaction between SREBP‐1 and ‐2 [25]. The former consists of two isoforms, SREBP‐1a and ‐1c and is the primary regulator of FFA synthesis, followed by TG synthesis, whereas the latter is the master regulator of cholesterol synthesis. They share a common substrate, acetyl‐CoA (Ace‐CoA). Under conditions of cholesterol deprivation, SREBP‐2 suppresses SREBP‐1 via microRNA‐33, leading to increased utilization of Ace‐CoA for cholesterol synthesis; conversely, in cholesterol‐rich conditions, SREBP‐1 suppression is lifted to accelerate FFA synthesis [25]. We inferred that SLURP2 may supply more Ace‐CoA to SREBP‐2 by inhibiting SREBP‐1, thereby accelerating cholesterol synthesis (Figure 4). By contrast, our gene expression assay found no significant upregulation of *Srebf2* expression (Figure 2I). Furthermore, we could not examine the protein expression of SREBP‐2 quantitatively as there were neither adequate antibodies for simple Western blotting, nor sufficient protein quantities for general Western blotting. However, the ratio of SREBP‐2‐positive adipocytes in the dermal adipocytes was found to be significantly higher in the SLURP2 group than in the control group (Figure 2J). This result indicates that SLURP2 is involved in either the regulation or stimulation of SREBP‐2. Accordingly, although it remains unclear whether SLURP2 directly regulates SREBP‐2, our study suggests that SLURP2 may accelerate cholesterol synthesis by suppressing SREBP‐1, followed by the stimulation of SREBP‐2, to enlarge adipocytes through the mechanism as aforementioned. (Figure 4).

Despite the above findings, mouse *SLURP2*‐deficient mice showed no significant difference in plasma TG levels compared with wild‐type mice. Based on our results, SREBP‐1 was assumed to be upregulated in mouse *SLURP2*‐deficient mice, which would result in a significant increase in TG levels. However, the cause of this phenotype remains unclear. While SREBP‐1a is expressed ubiquitously, SREBP‐1c is predominantly expressed in hepatocytes and adipocytes [28]. In adipocytes, SREBP‐1c is not involved in FFA synthesis [29]. Moreover, while transgenic mice expressing nuclear SREBP‐1a exhibit an increase in plasma FFA, those expressing nuclear SREBP‐1c show normal levels [30]. Accordingly, these studies may support the findings of normal serum TG levels in mouse *SLURP2*‐deficient mice.

A high level of cholesterol is essential for adipocyte expansion, which requires expansion of both the plasma membrane (PM) and the lipid droplet membrane. We hypothesized that SLURP2 may stimulate cholesterol synthesis to expand the adipocyte PM and lipid droplet membrane to promote the uptake of TG. As shown in a previous study, mouse *SLURP2*‐deficient mice exhibit distorted oval‐shaped dermal adipocytes in the ear, whose PM tension seems to be weak, compared with the spherical adipocytes observed in wild‐type mice [12]. This is inferred to result from a deficiency of cholesterol in the adipocyte PM. Given that mouse *SLURP2*‐deficient mice exhibit a reduction in adiposity, we suggest that SLURP2 may enlarge not only dermal adipocytes but also adipocytes in other locations in the same manner.

Meanwhile, our immunohistochemical analyses revealed that SLURP2 increases CAV‐1 protein expression in dermal adipocytes compared with control (Figure S3). Cav‐1 is more highly expressed in mature adipocytes than in preadipocytes [16]. Thus, this result indicates that there are more mature adipocytes in rSLURP2‐ than control‐injected skin. However, the inverse development of CAV‐1 and SLURP2 was shown in psoriasis. Indeed, a decrease in CAV‐1 expression has been reported in the epidermis of psoriatic lesions and imiquimod‐induced psoriasis‐like mouse model [31, 32]. Furthermore, Roelandt et al. have suggested that the downregulation of CAV‐1 could contribute to the hyperproliferation of epidermal keratinocytes, which is characteristic of psoriasis [33]. In addition, they have reported an increase in lipid raft formation, invagination of localized assemblies of cholesterol and sphingolipids within the PM, parallels with the epidermal hyperplasia in psoriasis as well as *cav‐1* ^−/−^mice [33]. Given that invagination morphology depends on the presence of cholesterol [33], an increase in lipid raft requires high cholesterol levels. Our results suggest that the upregulation of SLURP2 stimulates cholesterol synthesis in epidermal keratinocytes and contributes to the increase in lipid raft formation. The inverse development of Cav‐1 and SLURP2 may be connected in the epidermis of psoriasis.

Next, we found that SLURP2 induces IL‐23‐producing macrophages in the dermal adipose tissues (Figure 3B,C). Although Cherneyavsky et al. reported the anti‐inflammatory effect of SLURP2 in U937 macrophages [11], we demonstrated that SLURP2 exerts a pro‐inflammatory effect. Their results and ours indicate that SLURP2 exerts two opposing effects. A previous study by Lyukmanova et al. explained the cause of this phenomenon; they showed that SLURP2 alters ACh‐evoked currents at AChR in a concentration dependent manner. Specifically, while it maintains the cholinergic anti‐inflammatory pathway at lower concentrations, it inhibits α4β2 and α7 nAChRs (both of which are key receptors in that pathway) at higher, micromolar‐level concentrations (IC_50_ approximately 1.7 μM, > 3 μM, respectively). In particular, the inhibition of α4β2 nAChR has been shown to be concentration dependent. Moreover, α7 nAChR exerts a priming effect that reinforces its anti‐inflammatory function at very low concentrations (30 nM) [8]. Although Chernyavsky et al. used 1.1 nM (0.01 μg/mL) rSLURP2 [11], we used 1.1 μM (10 ng/μl). Their results and ours align with those of Lyukmanova et al. [8]. Our concentration was considered sufficient to inhibit α4β2 nAChR. Furthermore, they showed that SLURP2 binds to an antagonist site of α7 nAChR [8]. Suzuki et al. identified suppression of α4β2 nAChR expression in M1 macrophages and demonstrated the relationship between macrophage polarization and nAChR expression [34]. In addition, inhibition of α7 nAChR has been shown to induce M1 polarization [35]. Although we could not show M1 polarization, their studies support that SLURP2 induces the IL‐23‐producing macrophages (which include M1 macrophages) by suppressing α4β2 nAChR or binding to an antagonist site on α7 nAChR.

Moriwaki et al. have demonstrated that SLURP2 is regulated by the IL‐22/Signal transducer and activator of transcription 3 (Stat3) axis [36]. Stat3 is a key transcription factor involved in the pathogenesis of psoriasis [37]. IL‐22 is a crucial cytokine in psoriasis which, similar to SLURP2, is expressed in psoriatic lesions but not in non‐lesions or healthy skin. This cytokine is produced by Th17 cells and binds to the IL‐22 receptor on epidermal keratinocytes, leading to the secretion of SLURP2 by these cells. Upregulation of SLURP2 in psoriatic lesions is therefore considered to result from IL‐22 overexpression.

In conclusion, the present study demonstrated that SLURP2 exerts both metabolic and proinflammatory effects. Our results suggest that dermal adipose tissue may be a source of IL‐23 in psoriatic lesions. In addition, when combined with the previous study, our findings revealed that SLURP2 is involved in cholesterol synthesis. We thus propose that SLURP2 may serve as a link between psoriasis and metabolic syndrome.

## Author Contributions

H.T.: conception and design of the research study, experimental execution, data analysis and interpretation and manuscript writing. T.M.: data interpretation, manuscript review and revision.

## Ethics Statement

All animal experimental protocols were approved by the Animal Experimentation Committee of Tokai University. (approval numbers: #211095, December 24, 2021; #222027, April 1, 2022; #233009, April 1, 2023; and #244013, April 19, 2024).

## Conflicts of Interest

The authors declare no conflicts of interest.

## Supporting information


**Figure S1:** Statistical comparison among recombinant human SLURP2‐Fc (S), recombinant human IgG1 Fc (F) and PBS (P) for adipocyte area (a) and IL‐23‐producing macrophage ratio (b) by Friedman test followed by Dunn's multiple comparison. Abbreviations: SLURP2‐Fc, secreted Ly‐6/uPAR related protein 2 fused with Fc; IgG1, immunoglobulin G1; PBS, phosphate‐buffered saline.
**Figure S2:** (A) Representative images (×100) of H&E‐stained sections of dermal adipose tissue in rSLURP2 or control‐injected back skin for 7 days. Anagens I and II were occupied in dermis. There were no HFs in dermal adipose tissues. Scale bar; 100 μm. (B) Representative images (×200) and (C) (×400) of H&E‐stained sections of dermal adipose tissue. scale bars; (B) 50 μm, (C) 20 μm. Abbreviations: H&E, haematoxylin and eosin; rSLURP2, recombinant secreted Ly‐6/uPAR related protein 2; HF, hair follicle.
**Figure S3:** Comparison of Cav‐1 expression in dermal adipocytes. (A) Representative images (×200) of Cav‐1 immunohistochemical stained sections of dermal adipose tissue in rSLURP2 or control‐injected skin. scale bar; 50 μm. (B) Comparison of mean Cav‐1 staining intense score between SLURP2 and control groups. Data were analysed by Wilcoxon matched‐pairs signed‐ranks test . *p < 0.05, versus control. Abbreviations: Cav‐1, caveolin‐1; rSLURP2, recombinant secreted Ly‐6/uPAR related protein 2.
**Table S1:** Number of dermal adipocytes for area measurement. Abbreviations: SLURP2, secreted Ly‐6/uPAR related protein 2; PBS, phosphate‐buffered saline.

## Data Availability

The data that support the findings of this study are available from the corresponding author upon reasonable request.
