# Supplementary material for: SLURP2 Enlarges Adipocytes and Induces IL‐23‐Producing Macrophages in Murine Dermal Adipose Tissue
Source: Exp Dermatol. 2026 Jul 6;35(7):e70304. doi: 10.1111/exd.70304 (PMC13334338; doi:10.1111/exd.70304)
Supplement: Supplementary file 1 — Figure S1: Statistical comparison among recombinant human SLURP2‐Fc (S), recombinant human IgG1 Fc (F) and PBS (P) for adipocyte area (a) and IL‐23‐producing macrophage ratio (b) by Friedman test followed by Dunn's multiple comparison. Abbreviations: SLURP2‐Fc, secreted Ly‐6/uPAR related protein 2 fused with Fc; IgG1, immunoglobulin G1; PBS, phosphate‐buffered saline. Figure S2: (A) Representative images (×100) of H&E‐stained sections of dermal adipose tissue in rSLURP2 or control‐injected back skin for 7 days. Anagens I and II were occupied in dermis. There were no HFs in dermal adipose tissues. Scale bar; 100 μm. (B) Representative images (×200) and (C) (×400) of H&E‐stained sections of dermal adipose tissue. scale bars; (B) 50 μm, (C) 20 μm. Abbreviations: H&E, haematoxylin and eosin; rSLURP2, recombinant secreted Ly‐6/uPAR related protein 2; HF, hair follicle. Figure S3: Comparison of Cav‐1 expression in dermal adipocytes. (A) Representative images (×200) of Cav‐1 immunohistochemical stained sections of dermal adipose tissue in rSLURP2 or control‐injected skin. scale bar; 50 μm. (B) Comparison of mean Cav‐1 staining intense score between SLURP2 and control groups. Data were analysed by Wilcoxon matched‐pairs signed‐ranks test . *p < 0.05, versus control. Abbreviations: Cav‐1, caveolin‐1; rSLURP2, recombinant secreted Ly‐6/uPAR related protein 2. Table S1: Number of dermal adipocytes for area measurement. Abbreviations: SLURP2, secreted Ly‐6/uPAR related protein 2; PBS, phosphate‐buffered saline. [file EXD-35-e70304-s001.docx]

**Supplementary Figures & Table**

**Figure S1**

Statistical comparison among recombinant human SLURP2-Fc (S), recombinant human IgG1 Fc (F) and PBS (P) for adipocyte area (a) and IL-23-producing macrophage ratio (b) by Friedman test followed by Dunn's multiple comparison.

**Abbreviations**: SLURP2-Fc, secreted Ly-6/uPAR related protein 2 fused with Fc; IgG1, immunoglobulin G1; PBS, phosphate-buffered saline.

**Figure S2**

(A) Representative images (x100) of H&E-stained sections of dermal adipose tissue in rSLURP2 or control-injected back skin for 7 days. Anagens I and II were occupied in dermis. There were no HFs in dermal adipose tissues. Scale bar; 100μm. (B) Representative images (x200) and (C) (x400) of H&E-stained sections of dermal adipose tissue. scale bars; (B) 50μm, (C) 20μm.

**Abbreviations**: H&E, hematoxylin and eosin; rSLURP2, recombinant secreted Ly-6/uPAR related protein 2; HF, hair follicle.

**Figure S3**

Comparison of Cav-1 expression in dermal adipocytes.

(A) Representative images (x200) of Cav-1 immunohistochemical stained sections of dermal adipose tissue in rSLURP2 or control-injected skin. scale bar; 50μm.

(B) Comparison of mean Cav-1 staining intense score between SLURP2 and control groups. Data were analyzed by Wilcoxon matched-pairs signed-ranks test. **P* < 0.05, versus control.

**Abbreviations**: Cav-1, caveolin-1; rSLURP2, recombinant secreted Ly-6/uPAR related protein 2.

**Table S1. Number of dermal adipocytes for area measurement**

|  | 1 | 2 | 3 | 4 | 5 |
| --- | --- | --- | --- | --- | --- |
| PBS (control) | 293 | 106 | 274 | 314 | 183 |
| SLURP2 | 169 | 139 | 461 | 438 | 202 |
| Fc | 244 | 95 | 33 |  |  |

**Abbreviations**: SLURP2, secreted Ly-6/uPAR related protein 2; PBS, phosphate-buffered saline.
